# Nanoalum Formulations Containing Aluminum Hydroxide and CpG 1018^TM^ Adjuvants: The Effect on Stability and Immunogenicity of a Recombinant SARS-CoV-2 RBD Antigen

**DOI:** 10.3390/vaccines11061030

**Published:** 2023-05-26

**Authors:** Sakshi Bajoria, Ozan S. Kumru, Jennifer Doering, Katherine Berman, Greta Van Slyke, Anneka Prigodich, Sergio A. Rodriguez-Aponte, Harry Kleanthous, J. Christopher Love, Nicholas J. Mantis, Sangeeta B. Joshi, David B. Volkin

**Affiliations:** 1 Department of Pharmaceutical Chemistry, Vaccine Analytics and Formulation Center, University of Kansas, Lawrence, KS 66047, USA; 2 Division of Infectious Diseases, Wadsworth Center, New York State Department of Health, Albany, NY 12208, USA; 3 Department of Biological Engineering, Massachusetts Institute of Technology, Cambridge, MA 02139, USA; 4 The Koch Institute for Integrative Cancer Research, Massachusetts Institute of Technology, Cambridge, MA 02139, USA; 5 Bill & Melinda Gates Foundation, Seattle, WA 98109, USA; 6 Department of Chemical Engineering, Massachusetts Institute of Technology, Cambridge, MA 02139, USA

**Keywords:** nanoalum, CpG 1018, adjuvant, nanoparticle, vaccine, formulation, stability, immunogenicity

## Abstract

Aluminum-salt vaccine adjuvants (alum) are commercially available as micron-sized particles with varying chemical composition and crystallinity. There are reports of enhanced adjuvanticity when the alum’s particle size is reduced to the nanometer range. Previously, we demonstrated that a recombinant receptor-binding domain (RBD)-based COVID-19 vaccine candidate (RBD-J; RBD-L452K-F490W) formulated with aluminum hydroxide (Alhydrogel^®^; AH) and CpG 1018™ (CpG) adjuvants induced potent neutralizing antibody responses in mice yet displayed instability during storage. In this work, we evaluated whether sonication of AH to the nanometer size range (nanoAH) could further enhance immunogenicity or improve storage stability of the above formulation. The addition of CpG to nanoAH (at mouse doses), however, caused re-agglomeration of nanoAH. AH-CpG interactions were evaluated by Langmuir binding isotherms and zeta potential measurements, and stabilized nanoAH + CpG formulations of RBD-J were then designed by (1) optimizing CpG:Aluminum dose ratios or (2) adding a small-molecule polyanion (phytic acid, PA). Compared with the micron-sized AH + CpG formulation, the two stabilized nanoAH + CpG formulations of RBD-J demonstrated no enhancement in SARS-CoV-2 pseudovirus neutralizing titers in mice, but the PA-containing nanoAH + CpG formulation showed improved RBD-J storage stability trends (at 4, 25, and 37 °C). The formulation protocols presented herein can be employed to evaluate the potential benefits of the nanoAH + CpG adjuvant combination with other vaccine antigens in different animal models.

## 1. Introduction

Aluminum-salt adjuvants (generically referred to as alum) are commonly employed as vaccine adjuvants due to their well-established safety profile and large-scale availability at a low cost [1]. Based on their long history of use in vaccines, alum adjuvants are considered the “gold standard” against which new adjuvants are benchmarked [2]. Alum adjuvants exert their immunopotentiation effect by multifaceted mechanisms, including retention of the antigen at the injection site, recruitment of immune cells at the injection site, enhanced antigen uptake, direct and indirect stimulation of dendritic cells (DCs), and induction of CD4+ T cell differentiation into T_H_2 cells [3]. Aluminum hydroxide (e.g., Alhydrogel^®^, AH) and aluminum phosphate (e.g., AdjuPhos^®^, AP) are two commercially available alum adjuvants that differ in their physical and chemical properties [4]. AH has an isoelectric point (pI) of ~11 and is composed of fibrous, poorly crystalline aluminum oxyhydroxide particles (approx. 4.5 × 2.2 × 10 nm in dimension), whereas AP has a pI of 4–5 and is composed of disc-shaped, amorphous aluminum hydroxyphosphate particles (approx. 50 nm in diameter) formed by replacing hydroxyls with phosphate [4]. As aqueous colloidal suspensions, both AH and AP form porous agglomerates in the size range of 1–20 µm [4].

Recent reports have demonstrated that reducing the particle size of alum can enhance and broaden its adjuvant activity [2]. Compared with micron-sized alum particles, nanometer-scale (nanoalum) particles are more readily taken up by antigen-presenting cells (APCs) and, therefore, can induce enhanced humoral responses as well as cell-mediated (T_H_1) CD8+ CTL responses [5]. Additional potential advantages of nanoalum adjuvants include higher antigen adsorption capacity (due to greater surface area), enhanced freeze–thaw stability, and the ability to be sterile-filtered [6]. Upon preparation and storage, however, nanoalum can re-agglomerate over time and, thus, requires careful control of its formulation composition and processing conditions to ensure adjuvant stability and reproducibility [7,8].

CpG 1018 (a TLR-9 agonist) adjuvant is a negatively charged 22-base oligonucleotide used in a licensed hepatitis-B vaccine (HEPLISAV-B^®^) [9] and in combination with an AH adjuvant in a SARS-CoV-2 receptor-binding domain (RBD) subunit COVID-19 vaccine [10,11]. CpG 1018 adjuvants induce strong T_H_1 responses and can synergize with alum to induce stronger immune responses and enhance the potency of vaccines [2]. In our previous work, we demonstrated that a recombinant RBD-based vaccine antigen (termed RBD-J; RBD-L452K-F490W [12]) formulated with AH and CpG 1018 adjuvants elicited potent neutralizing antibody responses in mice but demonstrated instability during storage under real-time (4 °C) and accelerated (25 and 37 °C) conditions [13]. We and others have demonstrated that RBD antigen alone does not generate notable neutralizing responses in mice [12,13,14]. In addition, we have shown that other aluminum-adjuvanted formulations (e.g., AP, AH, and AP ± CpG 1018) do not generate notable neutralizing antibody responses in mice [13]. Therefore, on the basis of these previous reports, in this work, we focused on only the AH + CpG 1018 formulation of RBD-J, and our goal was to evaluate whether reducing the micron-sized AH particles to nanometer scale (nanoAH) could further enhance the immunogenicity and/or improve the storage stability of the formulated RBD-J in the presence of CpG 1018 as an additional adjuvant.

To this end, we sonicated micron-sized AH to form nanoAH and characterized the preparation by transmission electron microscopy (TEM) and laser diffraction analysis. We then evaluated the interactions of CpG 1018 with nanoAH in terms of adsorptive capacity (Langmuir binding isotherms) and particle surface charge (zeta potential). Next, to prevent re-agglomeration of nanoAH observed in the presence of CpG 1018 at mouse doses, we stabilized nanoAH by saturating the surface of nanoAH with an additional repulsive electrostatic charge using two different approaches: (1) employing lower AH and higher CpG 1018 doses to increase the CpG 1018:Aluminum ratio (referred to as the ‘higher CpG:Al’ approach) or (2) adding a small-molecular-weight polyanion, phytic acid (PA), along with CpG 1018 (referred to as the ‘CpG + PA’ approach). We then characterized these stabilized nanoAH + CpG 1018 formulations of RBD-J using physicochemical (UV-Visible spectroscopy, DSC, and SDS-PAGE) and immunochemical (competition ELISA binding to ACE2 receptor) assays. Finally, to determine in vitro and in vivo effect(s) of AH particle size in this vaccine candidate, we compared the two stabilized nanoAH + CpG 1018-adjuvanted formulations of RBD-J with the untreated AH + CpG 1018-adjuvanted RBD-J formulation in terms of storage stability profiles and neutralizing antibody responses in mice.

## 2. Materials and Methods

### 2.1. Materials

RBD-J was produced and purified by Biological E. Limited (Hyderabad, India) and stored at −80 °C at 2.4 mg/mL. The frozen protein stock solution was thawed at room temperature for 30 min prior to use, and the RBD-J antigen was diluted to a target concentration of 0.1 mg/mL in a “histidine formulation buffer” containing 20 mM Histidine, 100 mM NaCl, and 0.02% PS80 (pH 6.5), as employed previously [13]. The Alhydrogel^®^ (AH) adjuvant (10 mg/mL Aluminum content, # vac-alu-250) was purchased from InvivoGen (San Diego, CA, USA). The CpG 1018™ adjuvant was obtained from Dynavax Technologies (Emeryville, CA, USA). Inositol hexaphosphate (phytic acid, PA) sodium salt hydrate was purchased from Sigma-Aldrich (St. Louis, MO, USA), and other reagents were sourced as described previously [13].

### 2.2. Methods

#### 2.2.1. Sonication of AH

Stock AH at 10 mg/mL (Aluminum content) was diluted 2-fold (3–4 mL final volume) in histidine formulation buffer and sonicated in a 15 mL conical tube using a sonic dismembrator (Fisherbrand^TM^ Model 505, Fisher Scientific, Hampton, NH, USA) equipped with microtip probe (Fisherbrand™, Fisher Scientific, Hampton, NH, USA) operated at 35% power for 5 min with pulse ON for 2 min and OFF for 1 min. This step was performed in an ice bath to prevent over-heating of AH during sonication. Sonicated AH sample with CpG 1018 at previous mouse dose was prepared by adding CpG 1018 to sonicated AH at the final concentration of 1.5 mg/mL AH, 0.6 mg/mL CpG 1018 in histidine formulation buffer.

#### 2.2.2. Preparation of NanoAH Formulations of RBD-J in the Presence of CpG 1018

NanoAH formulations at 0.1 mg/mL RBD-J, 1.25 mg/mL sonicated AH, and 1 mg/mL CpG 1018 (F_Higher CpG 1018: Al_) or 0.1 mg/mL RBD-J, 1.25 mg/mL sonicated AH, 0.6 mg/mL CpG 1018, and 0.45 mg/mL PA (F_CpG 1018 + PA_) were prepared in histidine formulation buffer. For this, freshly sonicated AH at 5 mg/mL was slowly added to CpG 1018 ± PA in the formulation buffer and mixed gently by pipetting up and down, followed by addition of RBD-J. Samples were mixed well by pipetting and stored on benchtop for 30 min at room temperature for adsorption. Stock solution of PA was prepared at 15 mg/mL (in ultrapure water) immediately before use. This approach of adding nanoAH to the CpG 1018 ± PA solution was expected to help ensure a more uniform distribution of polyanions on the nanoalum surface.

For storage stability and mouse immunogenicity studies, various adjuvanted formulations of RBD-J were prepared with untreated or sonicated AH using the above methods. The prepared RBD-J formulations were shipped at 2–8 °C for mouse immunogenicity studies or incubated at different temperatures in storage stability studies (4, 25, and 37 °C for 3 months, 2 weeks, and 24 h, respectively), based on conditions described previously [13]. For mouse studies, prime and boost dose samples were prepared separately the day before injection.

#### 2.2.3. Laser Diffraction Particle Size Analysis Using Mastersizer 3000

A Mastersizer 3000 equipped with Hydro SV liquid dispersion unit (Malvern Panalytical, Malvern, UK) was used. Samples were injected into Hydro SV unit filled with ultrapure water, and measurements were recorded to achieve a ~10% obscuration rate with stirring at 1600 rpm. Two independent replicates were measured for each sample. The results were analyzed using the Mastersizer software (v3.63, Malvern Panalytical, Malvern, UK).

#### 2.2.4. TEM

The TEM method was adapted from elsewhere and optimized for our studies [15]. Samples were diluted to final concentration of 0.1 mg/mL AH in ultrapure water and loaded (5 µL) on a 300-mesh carbon-coated copper grid previously negative-glow-discharged for 30 s using EMS150R S sputter coater (Electron Microscopy Sciences, Hatfield, PA, USA). The excess liquid on the grid was wicked off after 1 min with a Kim wipe, washed with a drop of distilled water, dried using a Kim wipe, and examined using a Hitachi H8100 thermionic field emission transmission electron microscope operated at electron acceleration voltage of 200 kV. TEM images were captured using a normative and standardized electron dose on eucentric specimen stage and a constant de-focus value from the carbon-coated surfaces. Images were randomly acquired at different locations within the grid.

#### 2.2.5. Langmuir Adsorption Binding Isotherms with CpG 1018 and AH

Samples were prepared by adding 100 mcg AH to increasing amounts of CpG 1018 (0–400 mcg) in the histidine formulation buffer and mixed by gentle rotation on an end-over-end rotator for 30 min at room temperature. All samples were centrifuged at 4000× *g* for 5 min, and the supernatant was analyzed by UV-Visible spectroscopy (260 nm) to quantify the amount of unbound CpG 1018, as described previously [13]. To compare binding capacity of untreated vs. sonicated AH, 100 mcg untreated or sonicated AH was added to 100–800 mcg CpG 1018 in histidine formulation buffer. Samples were mixed and centrifuged, and the supernatant was analyzed for unbound CpG 1018 by the procedure described above. The data were fit to the linearized form of the Langmuir equation to calculate the maximum monolayer binding capacity of AH (Q_max_), as described elsewhere [16].

#### 2.2.6. Zeta Potential Measurements

The amount of 100 mcg AH was added to increasing amounts of CpG 1018 (0–200 mcg) in histidine formulation buffer, mixed by gentle rotation on an end-over-end rotator for 30 min at room temperature, and then diluted 100-fold in ultrapure water for zeta potential analysis using NanoBrook 90 Plus Zeta (Brookhaven Instruments, Holtsville, NY, USA) equipped with the zeta potential measurement electrode. Prior to measurement, the electrode was conditioned with saline, as per manufacturer’s recommendation. All measurements were conducted at 25 °C, and values for viscosity, refractive index, and dielectric constant were set to that for water. Brookhaven’s Particle Solutions software was used to obtain zeta potential values from electrophoretic mobilities using the Smoluchowski equation.

#### 2.2.7. RBD-J and CpG 1018 Characterization by SDS-PAGE, UV-Visible Spectroscopy, DSC, and ACE2 Competition ELISA

SDS-PAGE, UV-Visible spectroscopy, DSC, and ACE2 competition ELISA were performed by procedures described previously [13]. For UV-Visible spectroscopy in this work, Lunatic UV/Vis absorbance spectrometer (Unchained Labs, Pleasanton, CA, USA) instrument was used. For DSC analysis, samples were prepared at 2× concentrations of RBD-J, AH, CpG 1018, and PA to obtain thermograms with a good signal-to-noise ratio.

#### 2.2.8. Mouse Immunogenicity Studies

Mouse immunization (*n* = 8 per experimental group; *n* = 6 for control groups) and pseudovirus neutralization assay were performed as described previously [13]. Briefly, 50 µL of each formulation was subcutaneously administered to female BALB/c mice on study Days 0 and 21. Blood was collected via the submandibular vein on Days 21 and 35. For pseudovirus neutralization assay, pseudo-typed lentiviral reporter virus particles tagged with Renilla luciferase (Integral Molecular Inc., Philadelphia, PA, USA) were used with 293T-hsACE2 cells (Integral Molecular Inc., Philadelphia, PA, USA) in the absence and presence of mouse sera at indicated dilutions.

## 3. Results

### 3.1. Preparation, Characterization, and Stabilization of NanoAH + CpG 1018 Formulations

**Figure 1:**
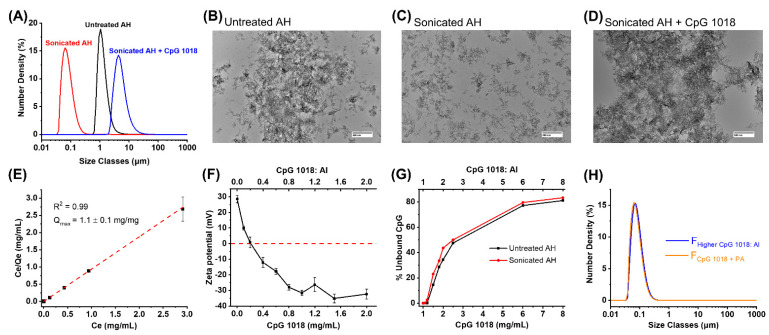
Preparation and characterization of stabilized nanoAH + CpG 1018-adjuvanted formulations of RBD-J by two different approaches (**A**) Representative particle size distribution profile of untreated (black) and sonicated AH before (red) and after (blue) addition of CpG 1018 as observed by laser diffraction; (**B**) Representative TEM images of untreated AH (scale bar = 500 nm); (**C**,**D**) Representative TEM image of sonicated AH (**C**) before and (**D**) after addition of CpG 1018 at doses used in previous mouse studies [13] (scale bar = 500 nm); (**E**) Linearized Langmuir adsorption isotherm for binding of CpG 1018 to untreated AH; (**F**) Zeta potential values of untreated AH in presence of increasing concentrations of CpG 1018; (**G**) Percentage of CpG 1018 remaining unbound to untreated vs. nanoAH at CpG 1018 concentrations greater than Q_max_ of untreated AH; (**H**) Representative particle size distribution profiles analyzed by laser diffraction of stabilized (nanoAH + CpG 1018)-adjuvanted formulations of RBD-J prepared using the “higher CpG:Al approach” (F_Higher CpG 1018: Al_) or the “CpG + PA” approach (F_CpG 1018 + PA_); see text for details of formulation protocols.

Particle size distribution and morphology of AH before and after sonication was determined by laser diffraction and TEM, respectively (Figure 1). Untreated AH displayed a distribution in particle sizes in the range of ~ 0.8–10 µm (Figure 1A). In comparison, sonicated AH showed a nearly 10-fold smaller particle size range of 30–300 nm (referred as ‘nanoAH’). When CpG 1018 was added to nanoAH at doses used in previous mouse studies [13], re-agglomeration to larger micron-sized particles (2–20 µm in diameter) was observed. By TEM analysis, untreated AH demonstrated large clusters (>1 µm) of fibrillar particles (Figure 1B), whereas sonicated AH contained significantly smaller (<500 nm) and well-dispersed particles (Figure 1C). Consistent with laser diffraction analysis, TEM images of sonicated AH in the presence of CpG 1018 (at mouse doses) showed re-agglomerated AH clusters that were larger than untreated AH particles (Figure 1D).

To assess the potential cause(s) of CpG-induced re-agglomeration of nanoAH particles, we performed a combination of Langmuir binding studies and zeta potential measurements to determine (1) the binding capacity of AH for CpG 1018 and (2) the effect of CpG 1018 binding on AH surface charge. For these experiments, we were able to analyze only untreated AH since sonicated AH re-agglomerated upon addition of low doses of CpG 1018 [17]. The maximum binding capacity (i.e., Q_max_) value was measured as 1.1 ± 0.1 mg of CpG 1018 per mg of AH (i.e., CpG:Al = ~1) (Figure 1E). When increasing concentrations of CpG 1018 were added to AH, the zeta potential values of AH decreased from approx. +30 mV to −30 mV and then leveled off as the Q_max_ was reached (Figure 1F). To evaluate whether decreasing the particle size of AH from micron to nanometer size range affected the maximum binding capacity for CpG 1018, we added excess CpG 1018 to untreated and sonicated AH (i.e., CpG:Al > 1) and measured the percentage of unbound CpG 1018 remaining in the solution (Figure 1G). No differences were observed, and both samples displayed a similar percentage of unbound CpG 1018 at each concentration tested. This result suggests that decreasing the AH particle size did not impact its maximum binding capacity for CpG 1018.

Since the amounts of AH and CpG 1018 used in our previous mouse studies (CpG:Al~0.4) was well below the Q_max_ value (CpG:Al~1), we sought to prevent re-agglomeration of nanoAH in the presence of CpG 1018 by more uniformly saturating the surface of nanoAH particles with negatively charged CpG 1018 by different approaches. First, we lowered the AH dose and increased the CpG 1018 dose in the formulation such that CpG:Al~0.8 (referred as the ‘higher CpG:Al’ approach). Second, we added the polyanion phytic acid (PA) along with CpG 1018 at lower doses (referred as the ‘CpG + PA’ approach). Using the above two approaches, stabilized nanoAH particles of nanoscale size (30–300 nm) were obtained in the presence of CpG 1018 as co-adjuvant [17]. The particle size distribution of the stabilized nanoAH + CpG 1018 was maintained in the nanometer range (30–300 nm) upon addition of the RBD-J antigen, and no re-agglomeration was observed (Figure 1H). The morphologies of nanoAH particles in the two stabilized nanoAH + CpG 1018-adjuvanted RBD-J formulations were indistinguishable and comparable to the morphology of freshly sonicated AH alone [17]. In summary, using either the ‘higher CpG:Al’ or ‘CpG + PA’ approaches, we successfully prevented re-agglomeration of nanoAH and prepared stabilized nanoAH formulations of RBD-J with CpG 1018 as a co-adjuvant.

### 3.2. Characterization of RBD-J Antigen Formulated with NanoAH and CpG 1018 Adjuvants

**Figure 2:**
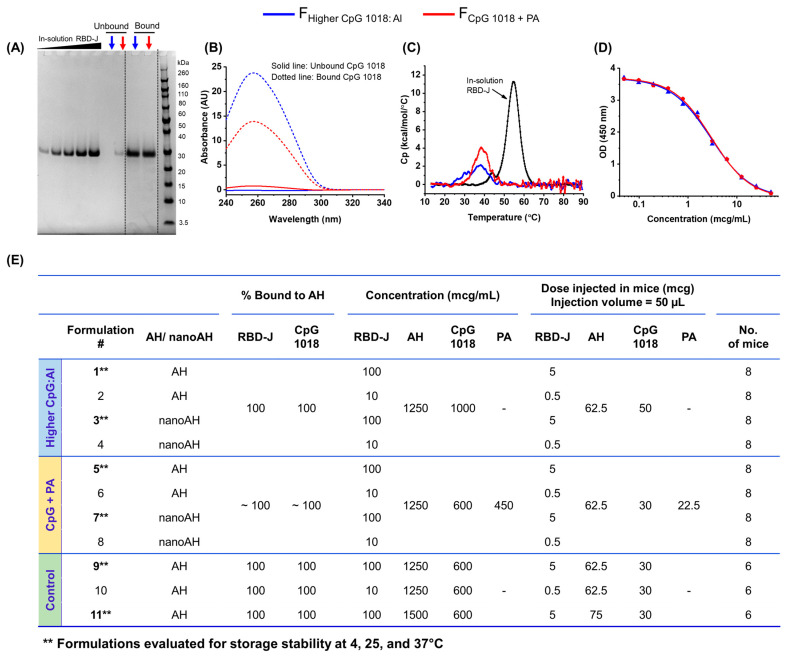
Characterization of stabilized nanoAH + CpG 1018-adjuvanted formulations of RBD-J prepared by two different approaches. (**A**) Representative reduced SDS-PAGE gel to quantify binding of RBD-J to nanoAH in the two formulations prepared using “higher CpG:Al” or “CpG + PA” approach; see text for details of formulation protocols; The dashed dividing lines on the gel indicate images of different parts of the same gel (densitometry readings and uncropped gel images can be found in Supplementary Materials). (**B**) Representative UV-Visible spectroscopy absorbance spectra of CpG 1018 either unbound (solid line) and bound (dotted line) to nanoAH in formulations prepared using “higher CpG:Al” (red) or “CpG + PA” (blue) approach; (**C**) Representative DSC thermograms of RBD-J in solution (black) and bound to nanoAH in formulations prepared using “higher CpG:Al” (red) or “CpG + PA” (blue) approach; (**D**) Representative ACE2 competition ELISA curve to determine ACE2-binding activity of RBD-J in nanoAH + CpG 1018 formulations prepared using “higher CpG:Al” (red) or “CpG + PA” (blue) approach; (**E**) Overview of various adjuvanted RBD-J formulations prepared to assess the effect of AH particle size on in vitro storage stability and in vivo mouse immunogenicity of RBD-J. nanoAH + CpG 1018-adjuvanted RBD-J formulations (prepared using “higher CpG:Al” or “CpG + PA” approach) were compared with formulations prepared by same approach but using micron-sized (untreated) AH. Note that all formulations listed above were evaluated for in vivo immunogenicity, while only formulations containing higher RBD-J doses (i.e., F1, F3, F5, F7, F9, and F11) were evaluated for in vitro storage stability (indicated by **).

The two stabilized nanoAH + CpG 1018-adjuvanted formulations of RBD-J were prepared as described above and characterized for the binding of RBD-J and CpG 1018 to nanoAH (using SDS-PAGE and UV-Visible spectroscopy, respectively), antigen conformational stability (using DSC), and antigen ACE2-binding activity (using ACE2 competition ELISA) (Figure 2). Quantitative analysis of the supernatant and pelleted fractions of the two adjuvanted RBD-J formulations by reduced SDS-PAGE showed that nearly 100% RBD-J was bound to nanoAH in both formulations (Figure 2A). Small amounts of unbound RBD-J were observed in the supernatant fraction of formulation prepared using the CpG + PA approach, but levels were below the estimated limit of quantitation (LOQ < 15%). UV-Visible spectroscopic analysis demonstrated that ~100% CpG 1018 was bound to nanoAH in both formulations (Figure 2B).

By DSC analysis, in-solution RBD-J (control) displayed a single major endothermic unfolding phenomenon, with a thermal melting temperature (Tm) value of ~55 °C, whereas RBD-J in the two stabilized nanoAH + CpG 1018 formulations displayed a prominently lower Tm value of ~38 °C (Figure 2C), indicating that RBD-J was significantly de-stabilized in the presence of nanoAH and CpG 1018. A major reduction in apparent enthalpy of unfolding (ΔH’) values of RBD-J (~94 kcal/mol in solution) was also observed in these two formulations (~26 and ~36 kcal/mol for the ‘higher CpG:Al’ and ‘CpG + PA’ formulations, respectively). Dose–response curves for ACE2-binding activity of RBD-J in the two stabilized nanoAH + CpG 1018 formulations, as observed by competitive ELISA, overlapped well, and no differences in ACE2 binding of RBD-J were observed (Figure 2D).

We then prepared 11 different adjuvanted RBD-J formulations to systematically assess the effect of AH particle size on in vitro storage stability and in vivo mouse immunogenicity of RBD-J in the presence of CpG 1018 at two different antigen doses, as listed in Figure 2E: stabilized nanoAH + CpG 1018 prepared by the ‘higher CpG:Al’ approach (formulations F1–F4), stabilized nanoAH + CpG 1018 prepared by the ‘CpG + PA’ approach (formulations F5–F8), along with three control adjuvanted RBD-J formulations (F9–F11). The three control formulations (all using micron-sized AH without sonication) were prepared to understand better the effect of (1) PA polyanion and (2) the change in dose ratios of AH and CpG 1018 and to (3) directly compare results from this work with our previously reported mouse study using RBD-J formulated with untreated AH + CpG 1018 [13].

### 3.3. Effect of AH Particle Size on In Vitro Stability Profiles of RBD-J Antigen in the Presence of CpG 1018

**Figure 3:**
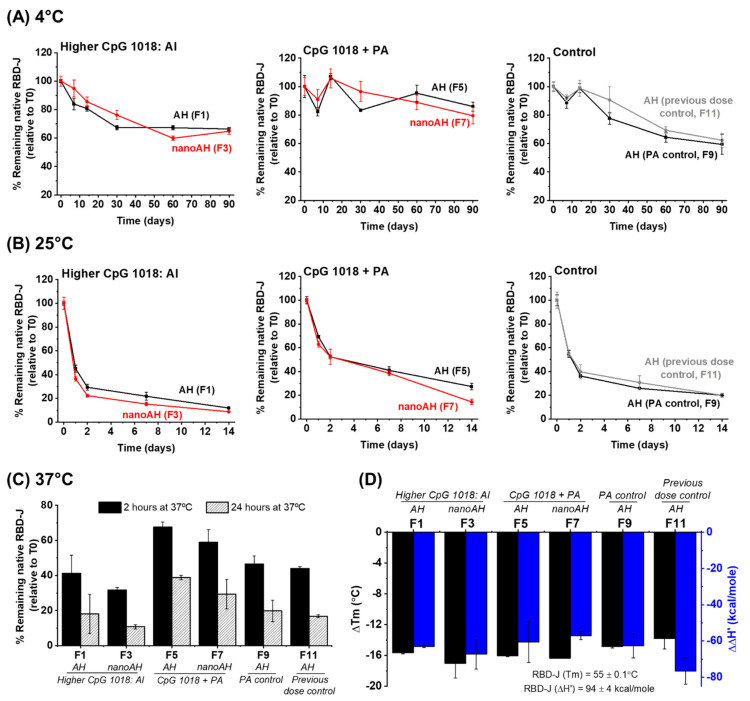
Effect of AH particle size on in vitro stability profiles and conformational stability values of RBD-J in the presence of CpG 1018. nanoAH + CpG 1018-adjuvanted RBD-J formulations were prepared using two approaches (“higher CpG:Al” or “CpG + PA”; see text for formulation protocols) and were compared with RBD-J formulations containing micron-sized untreated AH + CpG 1018. (**A**–**C**) Percentage of native RBD-J, as measured using ACE2 competition ELISA, remaining at each timepoint in each formulation during storage at (**A**) 4 °C, (**B**) 25 °C, and (**C**) 37 °C for 3 months, 2 weeks, and 24 h, respectively. The concentration of ACE2-binding native RBD-J at each time-point was normalized to values at T0 and plotted as mean ± SD for *n* = 4 replicates. Measured concentration of RBD-J in each formulation at T0 was close to target concentration of 100 mcg/mL [17]. (**D**) Conformational stability of RBD-J in the various formulations as determined using DSC. Bars represent averages and error bars represent ranges of data for *n* =2 replicates.

Stability studies were set up to compare RBD-J formulations with nanoAH + CpG 1018 (prepared using the two stabilization approaches described above) versus RBD-J formulated with micron-sized (untreated) AH + CpG 1018 adjuvants (at the high RBD-J dose levels only; see asterisk symbol in Figure 2E). Formulations were stored at 4, 25, and 37 °C for 3 months, 2 weeks, and 24 h, respectively, and analyzed for total protein by SDS-PAGE and ACE2-binding activity by competitive ELISA; the latter method was established previously as a stability-indicating assay [13] (Figure 3). By SDS-PAGE, no loss of protein was observed across the formulations, timepoints, or temperatures (>80% RBD-J remaining [17], a result that demonstrates that the total amount of RBD-J bound to AH in the various formulations remained unchanged during storage. The initial measured concentration of ACE2-binding ‘native’ RBD-J at T0 for each of the formulations was close to the target RBD-J concentration of 100 mcg/mL [17]. The concentration of ACE2-binding ‘native’ RBD-J at subsequent timepoints was normalized to the T0 values and reported as a percentage of the remaining native RBD-J antigen.

During storage at 4 °C over 3 months (Figure 3A), the RBD-J formulation prepared with nanoAH and CpG 1018 using the ‘higher CpG:Al’ approach (F3) demonstrated a stability profile similar to the formulation containing micron-sized untreated AH and CpG 1018 (F1), with both formulations displaying ~30% native antigen loss. For the nanoAH + CpG 1018 RBD-J formulation prepared using the ‘CpG + PA’ approach, no notable differences in ACE2-binding activity were exhibited compared with their micron-sized AH counterpart formulation (F5 vs. F7, respectively), with both formulations showing ~20% loss of native RBD-J. Furthermore, formulations prepared using the ‘CpG + PA’ approach demonstrated a consistent trend of improved RBD-J antigen stability (vs. formulations prepared using the ‘higher CpG:Al’ approach). In comparison with the control formulation F9 (micron-sized AH + CpG 1018 with no PA), which lost ~30% native RBD-J, the PA-containing formulation (F5) also showed improved stability profile. Finally, the control formulation F11, which was prepared with quantities of AH and CpG 1018 as used in a previous mouse study [13], showed a comparable stability profile to F9, indicating that higher AH doses did not affect the storage stability of RBD-J.

During accelerated storage stability results at 25 °C (Figure 3B) and 37 °C (Figure 3C), similar trends were observed. For example, at 25 °C, various AH + CpG 1018-adjuvanted RBD-J formulations lost ~70–80% native RBD-J within 2 weeks, with no differences noted between micron-sized vs. nanoAH formulations (F1 vs. F3 and F5 vs. F7). Similar to the results at 4 °C, CpG 1018 + PA formulations with either micron- or nano-sized AH (i.e., F5 and F7, respectively) displayed less loss of native antigen, indicating that the PA addition had a stabilizing effect on RBD-J at 25 °C. Similar stabilization trends of PA addition were observed in the various AH + CpG 1018-adjuvanted RBD-J formulations stored at 37 °C.

Finally, a DSC analysis was performed to evaluate the conformational stability of RBD-J in these same adjuvanted formulations. Results demonstrated significant and comparable decreases in Tm values and ΔH’ values of RBD-J in the adjuvanted formulations compared with RBD-J in solution (Figure 3D). The de-stabilizing effect of AH and CpG 1018 on conformational stability of RBD-J is consistent with our previous observations [13]. Under these conditions, the addition of PA did not provide any notable stabilization of the RBD-J, as measured by Tm and ΔH’ values, a result indicating the levels of AH and CpG 1018 present dominated the stability profile of the adjuvanted protein under stressed conditions of rapid temperature ramping.

### 3.4. Effect of AH Particle Size on In Vivo Immunogenicity of RBD-J Antigen in the Presence of CpG 1018

**Figure 4:**
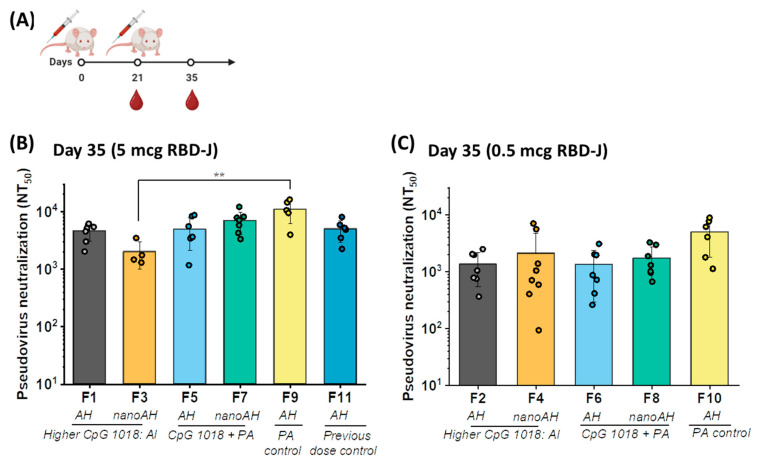
Effect of AH particle size on virus-neutralizing titers elicited by RBD-J in the presence of CpG 1018. (**A**) Female BALB/c mice were immunized by the subcutaneous route on Day 0 (prime) and Day 21 (boost). Serum was collected on Days 21 and 35. Pseudovirus neutralization titers (NT_50_) responses in mice groups vaccinated with nanoAH + CpG 1018-adjuvanted RBD-J formulations (prepared using ‘higher CpG:Al’ or ‘CpG + PA’ approach; see text for formulation protocols) were compared with RBD-J formulations containing untreated AH + CpG 1018 adjuvants. Pseudovirus neutralization titers (NT_50_) on Day 35 for mice groups immunized with (**B**) 5 mcg and (**C**) 0.5 mcg dose of adjuvanted RBD-J. Each circle represents an individual mouse. Bars represent group means (*n* = 8 mice per group for F1-F7; *n* = 6 mice per group for F9-F11) with standard deviations (sd). *p*-values were determined using Kruskal–Wallis test and post hoc Dunn’s multiple comparisons test (** *p* ≤ 0.01). Illustration in (**A**) was created with Biorender.com.

The same AH + CpG 1018-adjuvanted formulations of RBD-J evaluated during storage stability were also assessed for immunogenicity in mice. As shown schematically (Figure 4A), groups of mice were primed (Day 0) and then boosted (Day 21) with respective formulations, and sera were collected on Days 21 and 35 and analyzed for SARS-CoV-2 pseudovirus-neutralizing antibody titers. On Day 21, most mice elicited only low levels of pseudovirus-neutralizing antibody titers [17,18], a result observed in our previous study too [13]. On Day 35, however, all mice immunized with the various adjuvanted formulations containing 5 mcg RBD-J displayed robust neutralizing responses (Figure 4B). At a tenfold lower RBD-J dose (0.5 mcg), the adjuvanted formulations also elicited neutralizing antibody titers, albeit at lower levels (Figure 4C).

Surprisingly, no significant differences in neutralizing antibody titers were observed between groups vaccinated with micron-sized untreated AH + CpG 1018 vs. nanoAH + CpG 1018 containing RBD-J formulations (i.e., F1 vs. F3 and F5 vs. F7). Furthermore, the neutralizing titers in mice for the RBD-J formulations prepared using two stabilized nanoAH protocols were similar, i.e., ‘higher CpG:Al’ (F1 and F3) or ‘CpG + PA’ (F5 and F7). The control formulation (F9) formulated with micron-sized AH and CpG 1018 elicited neutralizing antibody titers similar to those of the F5 formulation, indicating that PA itself had no effect on the immunogenicity of RBD-J. Finally, the adjuvanted formulations of RBD-J induced levels of neutralizing antibody titers comparable to those of the control formulation (F11), which contained lower CpG 1018 and higher AH doses to match the formulation tested in our previous work [13]. In summary, no notable differences were observed in neutralizing antibody titers for RBD-J formulations containing micron-sized AH + CpG 1018 vs. nanoAH + CpG 1018, a result suggesting that reduction of AH particle size in the presence of CpG 1018 did not further enhance the humoral immune responses in mice.

## 4. Discussion

We previously reported potent neutralizing antibody responses in mice vaccinated with a recombinant SARS-CoV-2 RBD antigen (RBD-J) produced in a low-cost yeast expression system and formulated with AH and CpG 1018 adjuvants [13]. At the same time, several studies have reported that the adjuvant effect of alum can be enhanced by reducing its particle size ~10-fold (i.e., from micron to nanometer scale) [7,19,20,21]. For example, nanoalum can adsorb more antigen due to greater surface area and is better internalized by APCs, resulting in the induction of more potent immune responses [8]. In addition, there are also several reports that demonstrate that the addition of CpG 1018 as a second adjuvant to AH further enhances immune responses [19,20,21,22,23,24]. Therefore, drawing from these various literature reports, the major aim of this work was to assess the effect(s) of the combination of nanoAH and CpG 1018 adjuvants on the immunogenicity of RBD-J as well as its pharmaceutical properties, such as storage stability.

### 4.1. Stabilization of NanoAH + CpG 1018 Formulations

We prepared nanoAH from conventional AH using a “top-down” approach via sonication, which generated smaller fibrillar nanoparticles in the size range of 30–300 nm. The nanoAH (in the absence of CpG 1018 or antigen) showed no re-agglomeration when stored up to 3 weeks at 25 °C [17]. When CpG 1018 adjuvant was added to nanoAH at the mouse doses used in previous studies [13], rapid re-agglomeration to micron-sized particles (~2–20 µm) was observed. Reversing the sequence of compounding (e.g., the addition of sonicated AH to CpG 1018) or the use of more aggressive sonication conditions (e.g., longer sonication time and increased power strength) did not mitigate re-agglomeration of nanoAH upon CpG 1018 addition [17].

Since CpG 1018 is a negatively charged oligonucleotide with a phosphorothioate backbone, its binding to AH is driven by either an electrostatic or ligand exchange mechanism [9]. In our studies in the histidine formulation buffer, the maximum binding capacity (Q_max_) value of 1.1 mg CpG 1018 per mg AH (i.e., CpG 1018:Al~1) was observed. Since the CpG 1018:Al ratio (~0.4) employed in our previous mouse dose was significantly lower than Q_max_, we hypothesized that CpG 1018 caused cross-linking of unoccupied sites on AH particles, leading to its re-agglomeration. A similar hypothesis was made for re-agglomeration of AH observed at lower protein–antigen concentrations [15] (see below). Furthermore, the decrease in AH’s zeta potential values from ~ +30 mV to −10 mV in the presence of low doses of CpG 1018 (used in the mouse studies) could decrease stabilizing repulsive forces and, thus, lower the colloidal stability of nanoAH.

It has been reported in the literature that nanoalum preparations can re-agglomerate during longer-term storage, especially in the presence of an antigen, and stabilizers such as amino acids or polymers (e.g., PAA, PEG, and polyvinylpyrrolidone) are typically added to enhance the colloidal stability of nanoalum preparations [7,21,25,26,27]. Interestingly, a similar decrease in zeta potential of alumina particles has been reported at lower PAA concentrations, which resulted in an increase in alumina particle sizes [28]. Likewise, in another study, nanoAH was prepared by higher-pressure homogenization of conventional AH in the presence of varying amounts of PAA, and results showed larger hydrodynamic diameter values of nanoAH at lower PAA concentrations [29]. In terms of binding capacity, although nanoAH would be expected to demonstrate higher binding capacity due to smaller particle size and greater surface area, we did not observe any differences in the CpG 1018-binding capacity of untreated or nanoAH. The identical binding behavior of micron-sized vs. nanoAH particles in terms of antigen-binding capacity has been previously reported by other groups [15].

To prevent re-agglomeration of nanoAH in the presence of CpG 1018, we saturated the surface of nanoAH with negatively charged additives by two different approaches: (1) increasing CpG 1018 concentration and decreasing AH concentration (altering the CpG:Al ratio) or (2) adding a small-molecular-weight polyanion (phytic acid, PA) along with CpG 1018 at the mouse dose. The first approach contains a higher CpG 1018 concentration, so it is more challenging for use in mouse studies due to a lower permissible mouse dose of CpG 1018 (typically up to 50 mcg). To keep the CpG 1018 dose within permissible mouse dose limits, we reduced the AH concentration from 1500 mcg/mL (used in a previous study) to 1250 mcg/mL. For the second approach, we added inositol hexaphosphate (also called phytic acid, PA), a compound commonly found in plant tissues, which contains a high density of negative charges [30]. PA can interact with positively charged AH to saturate the AH surface with negative charge at lower CpG doses. Although PA is not found on the FDA’s list of inactive ingredients included in approved parenteral drug products, it has been tested previously in animal models and, thus, served as a “proof of concept” for this formulation approach. Interestingly, an initial evaluation of other charged additives (e.g., dextran sulfate and aspartic acid) did not prevent re-agglomeration of nanoAH [17]; however, it is possible that more extensive evaluations of charged compounds (different charge density, size, concentrations, etc.) may lead to alternatives to PA. Furthermore, since the two approaches used in our studies to prepare stable nanoAH work by electrostatic interaction mechanisms, future work is required to understand the effect of different formulation conditions (pH, ionic strength, presence of excipients, etc.) on the stabilizing potential of these two approaches.

### 4.2. Stability Profiles of RBD-J Antigen Formulated with NanoAH + CpG 1018

Due to differences in physicochemical properties of micron vs. nano- sized AH (e.g., size and surface area), differences in antigen–adjuvant interactions may be expected (e.g., binding capacity, conformational stability, and storage stability profiles). For RBD-J binding to AH, we previously observed that RBD-J binds essentially ~100% in the presence of CpG 1018 [13]. In this work, we similarly observed that RBD-J completely binds to AH in the presence of CpG 1018, independent of AH particle size. In terms of conformational stability (DSC) and storage stability (ACE2 competition ELISA) of the RBD-J antigen, similar stability trends were observed in the micron-sized AH + CpG 1018 vs. nanoAH + CpG 1018 formulations. This result suggests that the structural properties of the adsorbed RBD-J were unaffected by AH particle size in the presence of CpG 1018. The notable decline in conformational and storage stability of RBD-J in all formulations is consistent with our previous observations that both AH and CpG 1018 destabilize this antigen [13]. The PA-containing formulations displayed a trend of lower rate of degradation and improved RBD-J stability, indicating that the different ways to produce nanoAH + CpG 1018 formulations to minimize re-agglomeration of nanoAH can also impact the stability of the adsorbed antigen. The stabilizing effect of PA also shows promise that future formulation optimization work, including screening of stabilizing excipients, may help to overcome the de-stabilizing effect of AH and CpG 1018 on the RBD-J antigen to improve the storage stability of the vaccine candidate.

Finally, for the nanoAH + CpG 1018 formulations of RBD-J produced by the two different approaches, no changes in adjuvant particle size were observed throughout the duration of the study at 4, 25, or 37 °C [17]. Previous reports have shown that sterile PAA-stabilized nanoalum preparations can be manufactured using pharmaceutical equipment at larger scale and that the particles, albeit in the absence of antigen, showed no size growth when stored up to 1 year at 5 °C and 3 months at 25 and 37 °C [7]. Taken together, these results show the potential for GMP manufacturing of stabilized nanoalum adjuvants in vaccine formulations that may be stored long-term under refrigerated conditions or for limited times at ambient temperatures.

### 4.3. In Vivo Immunogenicity of NanoAH Formulations of RBD-J in the Presence of CpG 1018

To the best of our knowledge, only one other report has described the combination of nanoalum and CpG oligodeoxynucleotide as co-adjuvants, and this was performed in the context of developing a cancer vaccine candidate [27]. Using an acidified ovalbumin as a model antigen, Hou et al. observed significant increases in humoral and cellular immune responses in mice when PEG-stabilized aluminum hydroxide nanoparticles were co-formulated with two additional adjuvants, CpG 1826 and a double-stranded RNA-based adjuvant [27]. In general, adjuvant combinations have been successfully developed for prophylactic vaccines (e.g., AS01 and AS04 adjuvants from GSK) [31], and there is ongoing interest to employ adjuvant combinations with nanoparticles to improve the efficacy of cancer vaccine candidates [32].

In the mouse immunogenicity studies conducted here, AH + CpG 1018 vs. nanoAH + CpG 1018 formulations of RBD-J elicited similar levels of neutralizing antibody titers across the various formulations. It is possible that the presence of CpG 1018 as a co-adjuvant, at the doses used in our studies, may dominate in terms of directing the humoral immune response and/or, alternatively, interfere with nanoAH’s mechanism of action and negate any improved antibody responses. Furthermore, we focused our studies on generation of neutralizing antibody titers due to their correlation with clinical efficacy of COVID-19 vaccines [33], and, hence, neither total antibody responses nor the extent of improved T_H_1-based cellular responses were evaluated in this work. Nanoalum can augment cell-mediated immunity via CD4+ T_H_1 and CD8+ cytotoxic T cell responses [5,7,25,29]. It is possible that formulations prepared using nanoAH + CpG 1018 may perform better in terms of T cell-mediated cellular immune responses; however, these studies were beyond the scope of this work and will be of interest in the future.

There are conflicting reports on potentiation of immune responses comparing conventional micron-sized alum particles vs. nanoalum. For example, nanoalum has been reported to induce more potent immune responses, including both humoral (T_H_2) and cellular (T_H_1) responses [6], and to be more efficiently internalized by DCs and trafficked to the lymph node for T cell priming [21,34,35,36]. With specific vaccine candidates adsorbed to nanoalum, reports include stronger antigen-specific serum antibody responses against the *B. anthracis* subunit antigen [19] and hepatitis B surface antigen (HBsAg) [37]. In contrast, Vrieling et al. reported a comparable adjuvant effect of aluminum phosphate microparticles vs. nanoparticles with the diphtheria toxoid antigen, a result potentially attributed to agglomeration of nanoalum to micron-sized particles upon injection [26]. Although beyond the scope of this work, a more systematic, side-by-side comparison in mice of the immunogenicity of (1) RBD-J antigen alone, (2) antigen formulated with conventional alum (with and without CpG 1018), and (3) antigen formulated with nanoalum (with and without CpG 1018) is required to better understand the immune potentiation effects.

Some of the inconsistent observations with nanoalum adjuvants may be due to the variability in the method of preparation of the nanoalum adjuvant. Sun et al. demonstrated, by evaluating a library of aluminum oxyhydroxide nanorods, that immune responses by alum adjuvants can depend on their physicochemical properties, such as size, shape, crystallinity, and hydroxyl content [38]. Additionally, different approaches to prepare nanoalum (e.g., “top-down” vs. “bottom-up”), different processing equipment (e.g., ultrasonication vs. high-pressure homogenization), as well as varying formulation conditions (e.g., solution pH, stabilizers, compounding strategies, and storage time) may each result in differences in the nanoalum’s physicochemical properties [8,39]. For example, nanoAH stabilized using PAA elicited significantly higher cellular and humoral immune responses in mice compared with PEG-stabilized nanoAH [7]. The magnitude of the immune response was impacted by the molecular weight of PAA as well as the solution pH and the extent of PAA adsorption to AH nanoparticles [29]. Taken together, these literature reports demonstrate that the formulation protocols employed to prepare nanoalum are an important consideration for its effectiveness as a vaccine adjuvant.

## 5. Conclusions and Future Work

The two different formulation protocols described in this work to prepare stabilized nanoAH in the presence of CpG 1018 can be utilized in the future with other recombinant protein antigens to evaluate the potential utility of the nanoAH + CpG 1018 adjuvant combination in the development of stable and efficacious vaccine candidates. The first approach (‘higher CpG:Al’) employed higher CpG 1018 and lower AH doses, which, with some future optimization with other recombinant vaccine antigens, could be used at AH and CpG adjuvant doses typically employed for non-human primate and human studies [10,11]. The second approach (‘CpG + PA’) used lower doses of CpG 1018, which are more compatible for mouse studies. However, since phytic acid (PA) is not listed in the FDA’s inactive ingredient guide, future work to explore the use of other pharmaceutical polyanions based on this proof-of-concept approach is suggested. Furthermore, no improvements in neutralizing antibody titers in mice were observed with nanoAH vs. untreated conventional AH in the presence of CpG 1018; however, one of the nanoalum approaches (‘CpG + PA’) resulted in an improved trend of storage stability profile of RBD-J. Evaluating other recombinant protein antigens under similar conditions will allow for an assessment of the antigen-specific nature of these in vitro stability and in vivo immunogenicity observations. Although outside the scope of this work, it also will be of interest in the future to evaluate the ability of the nanoAH and CpG 1018 adjuvant combination to better potentiate cellular immune responses.

## Data Availability

The dataset generated and/or analyzed during the current study are available in the KU ScholarWorks repository, https://doi.org/10.17161/1808.32758. The data is also available with the corresponding author(s).

## Supplementary Materials

The following supporting information can be downloaded at: https://www.mdpi.com/article/10.3390/vaccines11061030/s1, Figure S1: Characterization of stabilized nanoAH + CpG 1018-adjuvanted formulations of RBD-J prepared by two different approaches.

## Abbreviations

Alhydrogel^®^, AH; Phytic acid, PA; CpG 1018^TM^, CpG; apparent enthalpy of unfolding, ΔH’; thermal melting temperature, Tm.
