# Supplementary material for: Nanoalum Formulations Containing Aluminum Hydroxide and CpG 1018TM Adjuvants: The Effect on Stability and Immunogenicity of a Recombinant SARS-CoV-2 RBD Antigen
Source: Vaccines (Basel). 2023 May 26;11(6):1030. doi: 10.3390/vaccines11061030 (PMC10303432; doi:10.3390/vaccines11061030)
Supplement: Supplementary file 1 [file vaccines-11-01030-s001.zip › vaccines-2344340-supplementary.pdf]

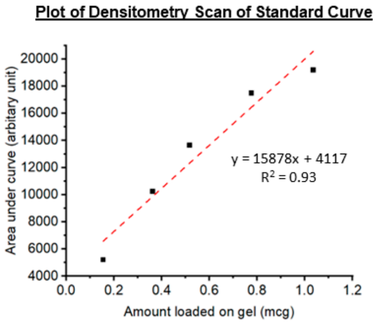

Gel Lane #	Sample	Densitometry Scan of Standard Curve and Test Samples<br>Area under curve (arbitrary units)	
1	In-solution RBD-J -1	5224	RBD-J standard curve
2	In-solution RBD-J -2	10240	
3	In-solution RBD-J -3	13646	
4	In-solution RBD-J -4	17479	
5	In-solution RBD-J -5	19194	
6	Higher CpG 1018:Al Unbound	2080	RBD-J + nanoAH + CpG 1018 samples
7	CpG 1018 + PA Unbound	3438	
8	Higher CpG 1018:Al Bound	19813	
9	CpG 1018 + PA Bound	18538	

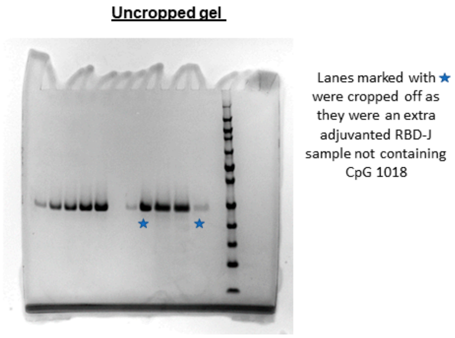

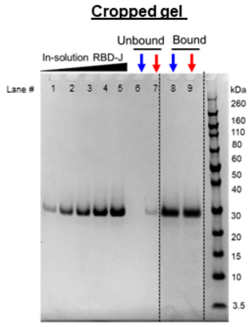

Figure S1. Characterization of stabilized nanoAH + CpG 1018-adjuvanted formulations of RBD-J prepared by two different approaches.
